# HSI2/VAL1 PHD-like domain promotes H3K27 trimethylation to repress the expression of seed maturation genes and complex transgenes in Arabidopsis seedlings

**DOI:** 10.1186/s12870-014-0293-4

**Published:** 2014-11-01

**Authors:** Vijaykumar Veerappan, Naichong Chen, Angelika I Reichert, Randy D Allen

**Affiliations:** Institute for Agricultural Biosciences, Oklahoma State University, 3210 Sam Noble Parkway, Ardmore, OK 73401 USA; Current address: Department of Biological Sciences, University of North Texas, Denton, TX 76203-5017 USA

**Keywords:** HSI2, VAL1, AGL15, DOG1, Transgene silencing, Seed-maturation, DNA methylation, Histone methylation, H3K27me3, 5-aza-2′-deoxycytidine

## Abstract

**Background:**

The novel mutant allele *hsi2-4* was isolated in a genetic screen to identify Arabidopsis mutants with constitutively elevated expression of a *glutathione S-transferase F8::luciferase* (*GSTF8::LUC*) reporter gene in Arabidopsis. The *hsi2-4* mutant harbors a point mutation that affects the plant homeodomain (PHD)-like domain in HIGH-LEVEL EXPRESSION OF SUGAR-INDUCIBLE GENE2 (HSI2)/VIVIPAROUS1/ABI3-LIKE1 (VAL1). In *hsi2-4* seedlings, expression of this *LUC* transgene and certain endogenous seed-maturation genes is constitutively enhanced. The parental reporter line (WT^*LUC*^) that was used for mutagenesis harbors two independent transgene loci, *Kan*^*R*^ and *Kan*^*S*^. Both loci express luciferase whereas only the *Kan*^*R*^ locus confers resistance to kanamycin.

**Results:**

Here we show that both transgene loci harbor multiple tandem insertions at single sites. Luciferase expression from these sites is regulated by the HSI2 PHD-like domain, which is required for the deposition of repressive histone methylation marks (H3K27me3) at both *Kan*^*R*^ and *Kan*^*S*^ loci. Expression of *LUC* and *Neomycin Phosphotransferase II* transgenes is associated with dynamic changes in H3K27me3 levels, and the activation marks H3K4me3 and H3K36me3 but does not appear to involve repressive H3K9me2 marks, DNA methylation or histone deacetylation. However, *hsi2-2* and *hsi2-4* mutants are partially resistant to growth inhibition associated with exposure to the DNA methylation inhibitor 5-aza-2′-deoxycytidine. HSI2 is also required for the repression of a subset of regulatory and structural seed maturation genes in vegetative tissues and H3K27me3 marks associated with most of these genes are also HSI2-dependent.

**Conclusions:**

These data implicate HSI2 PHD-like domain in the regulation of gene expression involving histone modifications and DNA methylation-mediated epigenetic mechanisms.

**Electronic supplementary material:**

The online version of this article (doi:10.1186/s12870-014-0293-4) contains supplementary material, which is available to authorized users.

## Background

Transition from seed maturation to seed germination and seedling development involves a complex network of genetic and epigenetic mechanisms that down-regulate the expression of seed maturation genes in seedlings [1–6]. Seed maturation is under the control of a group of transcriptional activators including LEAFY COTYLEDON1 (LEC1 [7]), LEC1-LIKE (L1L [8]), ABSCISIC ACID INSENSITIVE3 (ABI3 [9]), FUSCA3 (FUS3 [10]) and LEC2 [11], which are collectively called the “LAFL network” [3]. The B3-domain containing transcriptional repressors HIGH-LEVEL EXPRESSION OF SUGAR-INDUCIBLE GENE2 (HSI2) /VP1/ABI3-LIKE1 (VAL1) and its homolog HSI2-LIKE1 (HSL1)/VAL2 act redundantly to repress ectopic activation of embryonic traits during seed germination and seedling development by the “LAFL network” of transcriptional activators [12–16]. HSI2 was also shown to negatively regulate the expression of β-glucuronidase (GUS) or luciferase (LUC) reporters under the control of seed-maturation specific gene promoters in transgenic Arabidopsis seedlings and vegetative organs [17,18]. Since many of the genes repressed by HSI2 in vegetative tissues are involved in the maturation phase of seed development, including desiccation tolerance, knock-out *hsi2* mutant seedlings show enhanced tolerance to water deficit whereas the overexpression of *HSI2* resulted in hypersensitivity to desiccation stress [19]. Recently, it was shown that both *fus3* and *lec2* loss of function mutants can completely suppress the embryonic phenotype of *hsi2/hsl1* double mutant seedlings, while it is partially suppressed in *abi3*, *lec1* and *l1l* mutants [15]. These results indicate that HSI2 and HSL1 function redundantly to repress the expression of these regulatory genes in seedlings to prevent ectopic expression of embryonic traits during seed germination and vegetative development.

Developmental regulation of gene expression in plants is affected by chromatin mediated epigenetic mechanisms that include DNA methylation, chromatin remodeling, histone variants, and histone modifications [20,21]. DNA methylation at the 5′ position of cytosine plays important roles in transcriptional silencing of transposons, repeat sequences, transgenes and transcribed genes [22]. In addition to DNA methylation, histone modifications also play a vital role in the regulation of both transposons and transcribed genes in plants. Methylation of various lysine residues in the N-terminal tail of histone H3 is a well characterized epigenetic mechanism. In Arabidopsis, mono- (me1), di- (me2) or tri- (me3) methylation of histone H3 occurs mainly at lysine 4 (K4), lysine 9 (K9), lysine 27 (K27) and lysine 36 (K36) [23]. H3K4me3 and H3K36me3 are enriched on actively transcribed genes whereas H3K27me3 marks are associated with developmental repression of transcribed genes. H3K9me2/3 marks, which are associated with DNA methylation and small interfering RNAs (siRNAs), are enriched in heterochromatic regions known to be involved in transcriptional silencing of transposons, repeat sequences and transgenes [23,24].

HSI2 and HSL1 proteins were predicted to contain a PHD-like domain, a B3-DNA binding domain, a conserved cysteine and tryptophan residue-containing (CW) domain and an ethylene-responsive element binding factor-associated amphiphilic repression (EAR) motif [3,12,14,17,25,26]. Both CW and PHD protein domains are known to recognize methylated histone marks [23,27–30]. Hoppmann et al. [29] showed that the CW domain of HSI2 binds to H3K4me2 and H3K4me3 *in vitro* and, recently, it was reported that the HSL1 CW domain interacts with the histone deacetylase HDA19 to repress the “LAFL network” genes, including *LEC1* and *LEC2*, by promoting histone deacetylation and the addition of H3K27me3 marks [31]. However, molecular and epigenetic mechanisms underlying the HSI2 PHD-like domain-mediated regulation of gene expression remain to be elucidated.

Previously, we reported a novel *HSI2* allele, *hsi2-4*, in Arabidopsis that harbors a point mutation resulting in an amino acid substitution (C66Y) in the second zinc finger of the HSI2 PHD-like domain. The *hsi2-4* mutant seedlings that carry a *glutathione S-transferase F8::luciferase* (*GSTF8::LUC*) reporter gene showed constitutively elevated transgene expression [14]. In addition to the *LUC* transgene, HSI2 PHD-like domain is required for the non-redundant repression of several seed-maturation genes in seedlings. These genes include those that encode both regulatory factors such as FUS3, and AGAMOUS-Like 15 (AGL15) and structural proteins that include cupin family storage protein, oleosins, late-embryogenesis-related proteins and seed storage albumins. Moreover, seed-specific genes that are de-repressed in *hsi2-4* mutant seedlings are targets of H3K27me3 marks. Chromatin immunoprecipitation and quantitative PCR (ChIP-qPCR) analyses indicated that HSI2 PHD-like domain promotes H3K27me3 marks on transgene *GSTF8* promoter and *LUC* coding sequences to repress transgene expression in parental *GSTF8::LUC* reporter (WT^*LUC*^) seedlings [14]. Both WT^*LUC*^ and *hsi2-4*^*LUC*^ mutant plants harbor two independent transgene loci [14]. One locus, located on chromosome IV, confers kanamycin resistance and luminescence, whereas the second locus, which is on chromosome V, confers only luminescence. Based on kanamycin sensitivity, the chromosome IV and chromosome V loci were named as *Kan*^*R*^ and *Kan*^*S*^, respectively [14].

In this work, we show that HSI2 PHD-like domain represses *LUC* transgene expression from both *Kan*^*R*^ and *Kan*^*S*^ loci by promoting H3K27me3 marks but not DNA methylation and siRNA associated H3K9me2 marks. Expression of *Neomycin Phosphotransferase II (NPTII)* from the *Kan*^*R*^ locus is also partially suppressed in an HSI2-dependent mechanism. However, while our data indicate that DNA methylation and histone deacetylation are not involved in the transcriptional repression of transgene loci in WT^*LUC*^, the HSI2 PHD-like domain may play a role in the inhibition of seedling growth and development caused by DNA methylation inhibitor 5-aza-2′-deoxycytidine (5-azadC).

## Results

### Disruption of HSI2 PHD-like domain affects the expression of both *Kan*^*R*^ and *Kan*^*S*^ transgene loci

**Figure 1:**
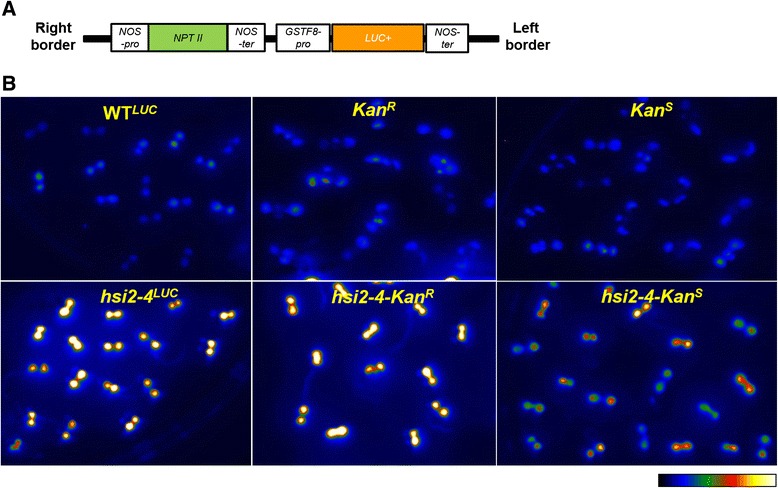
**Genomic structure of** ***GSTF8::LUC*** **transgene and luminescence imaging of WT** ^***LUC***^ **and** ***hsi2-4*** **mutant seedlings harboring either** ***Kan*** ^***R***^ **or** ***Kan*** ^***S***^ **transgene locus or both. A**. *GSTF8::LUC* transgene contain *neomycin phosphotransferase* (*NPTII*) coding sequences under the control of *nopaline synthase* (*NOS*) promoter and a modified *luciferase* (*LUC* ^*+*^ *)* coding sequences from firefly driven by *glutathione S-transferase F8* (*GSTF8*) promoter conferring kanamycin resistance and luminescence expression respectively in plants. The 3′ ends of both *NPTII* and *LUC* ^*+*^ coding sequences include *NOS* terminator sequences for transcriptional termination. **B**. Plants harboring either *Kan* ^*R*^ or *Kan* ^*S*^ transgene locus alone in the wild-type or in *hsi2-4* mutant background were obtained by crossing of either WT^*LUC*^ or *hsi2-4* ^*LUC*^ into Columbia-0 wild-type and homozygous lines were identified in F_2_ and F_3_ generations. Five days old seedlings of various genotypes grown on Murashige and Skoog media plates were imaged using cooled CCD camera after spraying with the substrate luciferin. Pseudocolor image indicates luminescence intensity from lowest (blue) to highest (white).

The *GSTF8::LUC* reporter construct contains a *GSTF8* promoter sequence that controls the transcription of a *luciferase* expression cassette, along with an *NPTII* gene under control of the *nopaline synthase* promoter and terminator sequences, which confers kanamycin resistance in plants (Figure 1A). The parental WT^*LUC*^ reporter line harbors two independent transgene insertion sites, *Kan*^*R*^ and *Kan*^*S*^. The *Kan*^*R*^ locus was mapped to chromosome IV, while the *Kan*^*S*^ locus is located on chromosome V (Table 1) [14]. Active luciferase is expressed by both *Kan*^*R*^ and *Kan*^*S*^ loci, conferring a luminescent phenotype; however, only the *Kan*^*R*^ locus expresses *NPTII*; thus, plants that harbor only the *Kan*^*R*^ locus are resistant to kanamycin, while *Kan*^*S*^ plants are sensitive to this antibiotic.

**Table 1:** **Estimation of** ***LUC*** **copy number in Arabidopsis plants containing either** ***Kan*** ^***S***^ **or** ***Kan*** ^***R***^ **locus or both**

**Genotype**	**Chromosomal locations**	**Calculated** ***LUC*** **copy number**	**Estimated** ***LUC*** **copy number**
*Kan* ^*S*^	5	2.00 ± 0.33	2
*Kan* ^*R*^	4	4.17 ± 0.47	5
WT^*LUC*^	-	7.10 ± 0.33	7

Number of *LUC* copies was determined by absolute quantitative real-time PCR. Calibration curves were created using *pBI121-GSTF8::LUC* plasmid DNA as a template. A single copy gene, At5g47480, was used as an internal control for normalization of the data. WT^*LUC*^ plants contain two unlinked transgene insertion loci composed of multiple T-DNA insertions. While both insertions express LUC only one expresses NPTII and confers resistance to kanamycin. These loci were separated by genetic segregation to produce lines that are kanamycin sensitive (*Kan* ^*S*^ ) or kanamycin resistant (*Kan* ^*R*^). *LUC* copy numbers were calculated for the *Kan* ^*S*^, *Kan* ^*R*^ and WT^*LUC*^ Arabidopsis lines as described previously [32,33]. Genomic DNA from Col-0 wild-type plants was used as a negative control.

To estimate the number of *LUC* copies at both *Kan*^*R*^ and *Kan*^*S*^ loci, real-time quantitative PCR (qPCR) was performed using genomic DNA from WT^*LUC*^, *Kan*^*R*^ and *Kan*^*S*^ plants. Since both *Kan*^*R*^ and *Kan*^*S*^ loci confer luminescence expression, we used PCR primers that are specific to the *LUC* coding sequences to estimate the copy numbers. The results show that *Kan*^*S*^ plants contain 2 copies of *LUC* whereas the *Kan*^*R*^ locus harbors 5 *LUC* copies. Independent analysis of WT^*LUC*^ plants, which contain both *Kan*^*R*^ and *Kan*^*S*^ loci, showed seven copies of the *LUC* transgene (Table 1). Therefore, both *Kan*^*R*^ and *Kan*^*S*^ loci are complex and contain multiple copies of the *GSTF8::LUC* transgene.

Previously, we showed that disruption of the HSI2 PHD-like domain affects the expression of the *Kan*^*R*^ transgene locus [14] but the effect of this mutation on the *Kan*^*S*^ locus was not evaluated. Therefore, to further investigate whether the *Kan*^*S*^ transgene locus is also regulated by the HSI2 PHD-like domain mutation and investigate potential interactions between *Kan*^*R*^ and *Kan*^*S*^ transgene loci in WT^*LUC*^ and *hsi2-4*^*LUC*^ mutant plants, these two loci were separated by crossing plants of the WT^*LUC*^ reporter line and the *hsi2-4*^*LUC*^ mutant line into Col-0 wild-type Arabidopsis and subsequent selection for homozygous WT^*LUC*^ and *hsi2-4* lines that carry either the *Kan*^*R*^ or *Kan*^*S*^ reporter gene locus.

**Figure 2:**
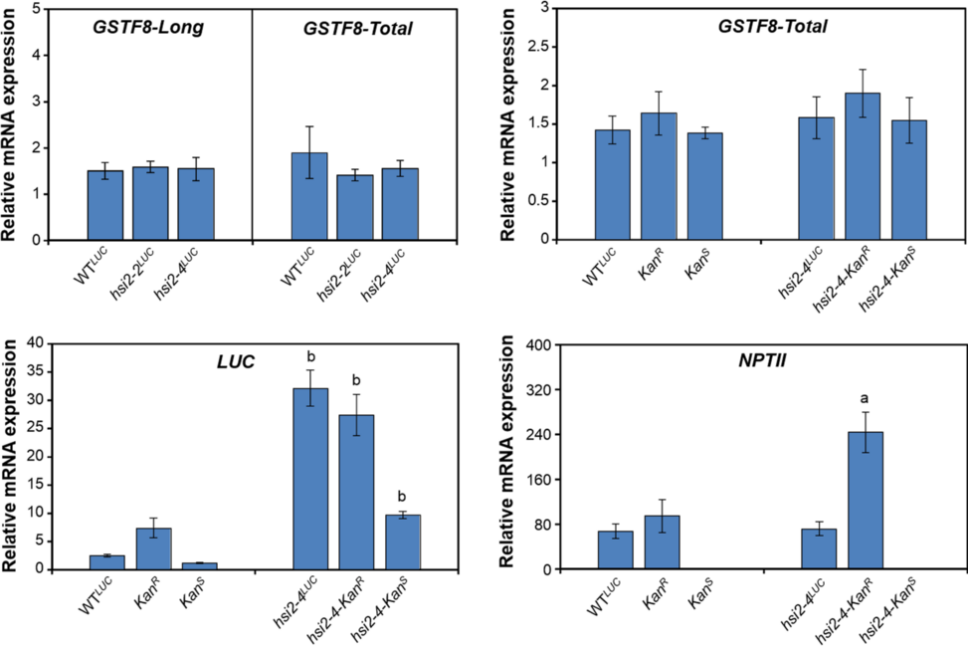
**Transcript levels of endogenous** ***GSTF8*** **and transgenes in WT** ^***LUC***^ **and** ***hsi2*** **mutants carrying either** ***Kan*** ^***R***^ **or** ***Kan*** ^***S***^ **transgene locus or both.** Real-time reverse transcription quantitative PCR was used to determine the relative transcript levels of endogenous *GSTF8*, *LUC* and *NPTII* genes in five day old seedlings of various genotypes. *GSTF8* produces two different transcripts with different fragment lengths by alternative start sites namely *GSTF8-Long* and *GSTF8-Short* [34]. Expression of *GSTF8-Total* represents transcripts from both *GSTF8-Long* and *GSTF8-Short* versions whereas *GSTF8-Long* expression level corresponds to *GSTF8-Long* transcript. *EF1α* was used for normalization. Data represent means (±SD) of two biological replicates with three technical replicates each. Significant differences in *LUC* transcript levels between the three luciferase reporter lines in the wild-type background and the respective *hsi2-4* mutant background, determined using two-tailed Student’s *t*-test assuming unequal variances, are indicated by letters (a = *p* < 0.001 and b = *p* < 0.0001).

Comparison of luciferase expression in seedlings homozygous for the isolated *Kan*^*R*^ and *Kan*^*S*^ transgene loci in the wild-type background showed that *Kan*^*R*^ seedlings had higher luminescence signals (Figure 1B) and steady state levels of *LUC* mRNA (Figure 2) than *Kan*^*S*^ seedlings. This is in agreement with the relative number of transgene copies at these loci. However, in spite of carrying more *luciferase* transgene copies than *Kan*^*R*^ seedlings, WT^*LUC*^ seedlings, showed significantly lower luminescence signal and *LUC* transcript levels. On the other hand, analysis of the expression of these transgenes in the *hsi2-4* background showed strongly enhanced *luciferase* expression in all of the lines and the relative levels of both luminescence signal and *LUC* transcripts corresponded with transgene copy number, with highest levels seen in *hsi2-4*^*LUC*^ seedlings and lowest levels in *hsi2-4-Kan*^*S*^ samples (Figures 1B and 2). This could indicate that, in a wild-type background, the presence of both the *Kan*^*R*^ and *Kan*^*s*^ loci may lead to stronger suppression of transgene expression but disruption of the HSI2 PHD-like domain affects the expression of *LUC* transgenes at both *Kan*^*R*^ and *Kan*^*S*^ loci similarly. Thus, the more complete HSI2-mediated repression of the *GSTF8::LUC* transgenes in WT^*LUC*^ plants results in stronger relative activation of their expression in the presence of the *hsi2-4* mutation.

Alternative transcriptional start sites of the endogenous *GSTF8* gene result in two different transcripts with different sizes: *GSTF8-Long* (*GSTF8-L*) and *GSTF8-Short* (*GSTF8-S*) [34]. To determine whether endogenous *GSTF8* expression is altered in *hsi2* mutant alleles, we performed qRT-PCR using various wild type lines (WT^*LUC*^, *Kan*^*R*^ and *Kan*^*S*^) and *hsi2* mutant lines (*hsi2-2*^*LUC*^, *hsi2-4*-*Kan*^*R*^, *hsi2-4-Kan*^*S*^ and *hsi2-4*^*LUC*^). *hsi2-2* is a loss-of-function mutant allele that carries a T-DNA insertion in the seventh exon of *HSI2* gene (SALK_088606) [12–14,17]. To obtain the *hsi2-2*^*LUC*^ line, *GSTF8::LUC* transgenes were introgressed into the *hsi2-2* mutant background by genetic crossing. Expression of *GSTF8-Total* (*GSTF8-T*) represents both *GSTF8-L* and *GSTF8-S* transcripts whereas *GSTF8-Long* expression represents *GSTF8-L* transcripts only. As shown in Figure 2, levels of endogenous *GSTF8-L* and *GSTF8-T* transcripts were not significantly affected in *hsi2-2*^*LUC*^ and *hsi2-4*^*LUC*^ plants and *NPTII* expression was not detected in the *Kan*^*S*^ reporter line, consistent with the kanamycin sensitivity of these plants. *NPTII* transcripts were expressed at similar levels in the WT^*LUC*^ and *hsi2-4*^*LUC*^ seedlings but expression of *NPTII* in *Kan*^*R*^ seedlings was responsive to the HSI2 PHD-like domain point mutation (Figure 2). Steady-state levels of *NPTII* transcripts from the *Kan*^*R*^ locus were about 3-fold higher in *hsi2-4* seedlings than in the wild-type background. Taken together, these results indicate that both the *GSTF8::LUC* and *NOS::NPTII* transgenes of the T-DNA cassette are partially suppressed by HSI2 in *Kan*^*R*^ seedlings; however the *NPTII* genes at the *Kan*^*S*^ locus may be fully silenced and/or contain loss-of-function mutations. Furthermore, in WT^*LUC*^ seedlings where the *Kan*^*S*^ locus is present, expression of both *LUC* and the *NPTII* genes at the *Kan*^*R*^ is more strongly suppressed.

### Luciferase expression is not affected by DNA methylation or histone deacetylation inhibitors

**Figure 3:**
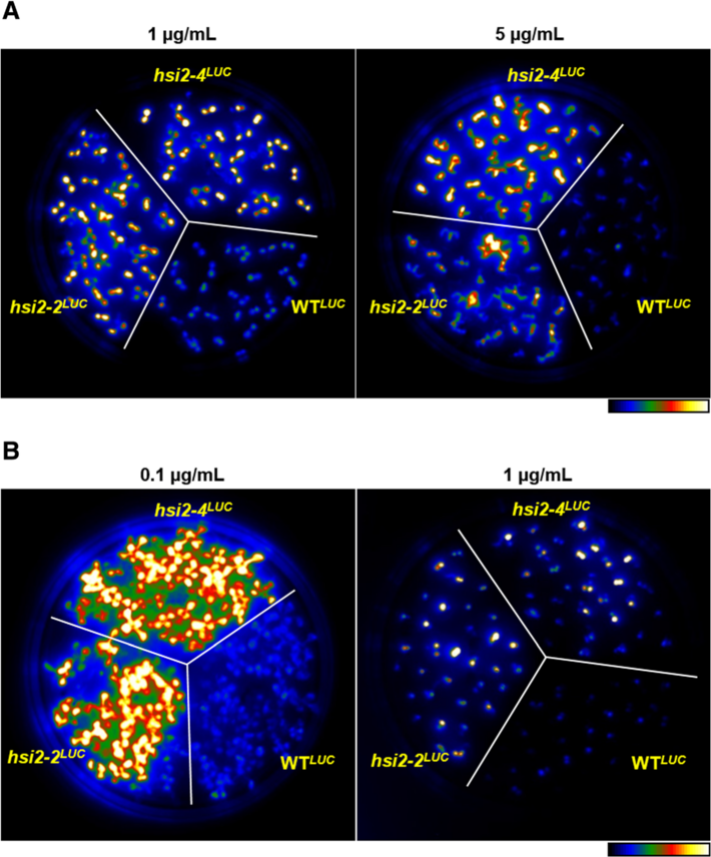
**Treatments of WT** ^***LUC***^ **,** ***hsi2-2*** ^***LUC***^ **and** ***hsi2-4*** ^***LUC***^ **seedlings with DNA methylation inhibitor 5-aza-2′-deoxycytidine (5-azadC) and histone deacteylase inhibitor Trichostatin A (TSA).** Seeds were germinated and grown vertically on media plates containing the indicated concentrations of either 5-azadC **(A)** or TSA **(B)**. Luminescence imaging of 10 days old seedlings was performed using cooled CCD camera after spraying with the substrate luciferin. Pseudocolor images show luminescence intensity from lowest (blue) to highest (white). The experiment was repeated with two technical replicates and representative images are shown.

Complex transgenes with tandem repeats in plants are often subjected to DNA methylation and histone deacetylation mediated transcriptional gene silencing [35]. If the *GSTF8::LUC* transgene loci in the WT^*LUC*^ plants are targets of DNA methylation, treatment of these seedlings with an inhibitor of DNA methylation should derepress the luminescence expression similar to that seen in *hsi2-4* seedlings. Previous reports showed that treatment with 5 μM/mL of the DNA methylation inhibitor 5-aza-2′-deoxycytidine (5-azadC) was effective in derepressing the transcriptional silencing of auxin-responsive ß-GUS reporter lines [35]. Treatment of Arabidopsis seedlings with 7 μM/mL 5-azadC also caused global changes in gene expression and derepression of silenced transgenes [36,37]. To investigate whether DNA methylation is involved in repressing *GSTF8::LUC* transgene expression, WT^*LUC*^, *hsi2-2*^*LUC*^ and *hsi2-4*^*LUC*^ seedlings were grown on media containing 5-azadC at various concentrations. Luminescence expression in WT^*LUC*^ seedlings was not affected at either 1 or 5 μM/mL 5-azadC concentrations (Figure 3A). Hence, DNA-methylation does not appear to be required for the repression of *LUC* expression in WT^*LUC*^ seedlings.

Histone deacetylation is known to regulate gene expression by transcriptional repression in eukaryotes [38]. Recently, it was reported that treatment of Arabidopsis seedlings with the histone deacetylase inhibitor trichostatin A (TSA) or down-regulation of two histone deacetylase genes, *HDA6* and *HDA19* by RNA interference resulted in derepression of the embryonic program in germinating seeds and seedlings [39]. Arabidopsis seedlings treated with TSA and histone deacetylase mutants mimic the phenotypes of *hsi2-2/hsl1*double mutant seedlings [12,13], indicating that HSI2- and HSL1-mediated repression of the embryonic program could involve histone deacetylation. HSL1 was shown to physically interact with HDA19 via its CW domain and disruption of HSL1 resulted in increased H3K4me3 and decreased H3K27me3 marks on genes that encode transcriptional activators involved in the embryonic program [31]. To test the effects of TSA on the luminescence expression of WT^*LUC*^ seedlings, WT^*LUC*^, *hsi2-2*^*LUC*^ and *hsi2-4*^*LUC*^ seedlings were grown on media containing 0.1 and 1 μg/mL TSA. Since higher concentrations of TSA resulted in severe growth retardation and developmental delay in all seedlings tested (Figure 3B), only, 0.1 and 1 μg/mL of TSA was used in these assays. Luminescence imaging data showed that treatments of WT^*LUC*^ seedlings with TSA did not affect their luminescence expression (Figure 3B), indicating that HSI2 PHD-like domain mediated repression of *LUC* transgene expression in WT^*LUC*^ seedlings is not dependent on TSA-sensitive histone deacetylation.

### *hsi2-2*^*LUC*^ and *hsi2-4*^*LUC*^ mutant seedlings are partially resistant to DNA methylation inhibitor 5-azadC induced growth inhibition

**Figure 4:**
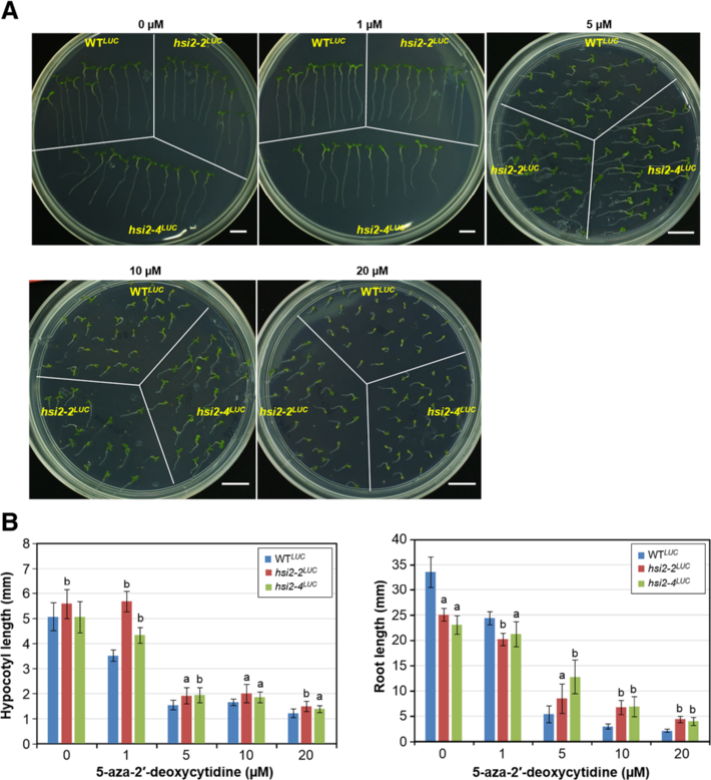
**Effects of DNA methylation inhibitor 5-aza-2′-deoxycytidine (5-azadC) on the growth and development of WT** ^***LUC***^ **,** ***hsi2-2*** ^***LUC***^ **and** ***hsi2-4*** ^***LUC***^ **seedlings.** Seeds were germinated vertically on media plates containing various concentrations of 5-azadC and pictures were taken 7 days after germination. **A**. Morphology of seedlings. **B**. Measurements of hypocotyl and root growths. Hypocotyl and root lengths were measured using ImageJ software. Data represent mean values (±SD) from 10 seedlings. The experiments were repeated with two technical replicates. Letters indicate significant differences between WT^*LUC*^ and *hsi2-2* ^*LUC*^ or *hsi2-4* ^*LUC*^ at each time point (a = *p*< 0.005, b = *p*< 0.0001).

We noticed that the growth and development of WT^*LUC*^ seedlings on plates that contained 5 μM/mL 5-azadC was more strongly inhibited than *hsi2-2*^*LUC*^ and *hsi2-4*^*LUC*^ mutant seedlings (Figure 4A). To further characterize the effects of 5-azadC on hypocotyl and root growth, WT^*LUC*^, *hsi2-2*^*LUC*^ and *hsi2-4*^*LUC*^ seeds were germinated on media containing 0, 1, 5, 10 and 20 μM 5-azadC. After 7 days of incubation on 5-azadC-containing media, all seedlings showed dose-dependent inhibition of growth and development. However, the most severe effects were seen with WT^*LUC*^ seedlings whereas the growth of *hsi2-2*^*LUC*^ and *hsi2-4*^*LUC*^ mutant seedlings was less inhibited (Figure 4A). While WT^*LUC*^ seeds germinated when incubated on media containing 20 μM 5-azadC, subsequent root growth and cotyledon development was almost completely abrogated while both and *hsi2-4*^*LUC*^ and *hsi2-2*^*LUC*^ mutant seedlings continued to grow and develop, albeit slowly, under these conditions (Figure 4A and B). Comparative measurements of hypocotyl and root growth indicated that *hsi2-2*^*LUC*^ and *hsi2-4*^*LUC*^ mutant seedlings were about one half as sensitive to 5-azadC-dependent inhibition as WT^*LUC*^ seedlings at 5, 10 and 20 μM 5-azadC treatments (Figure 4B). These data indicate that, although 5-azadC does not affect the HSI2-dependent suppression of luciferase expression in WT^*LUC*^ plants, HSI2 does affect sensitivity to 5-azadC-dependent inhibition of seedling development.

### *LUC* and *NPTII* transgene expression is associated with changes in histone methylation marks

**Figure 5:**
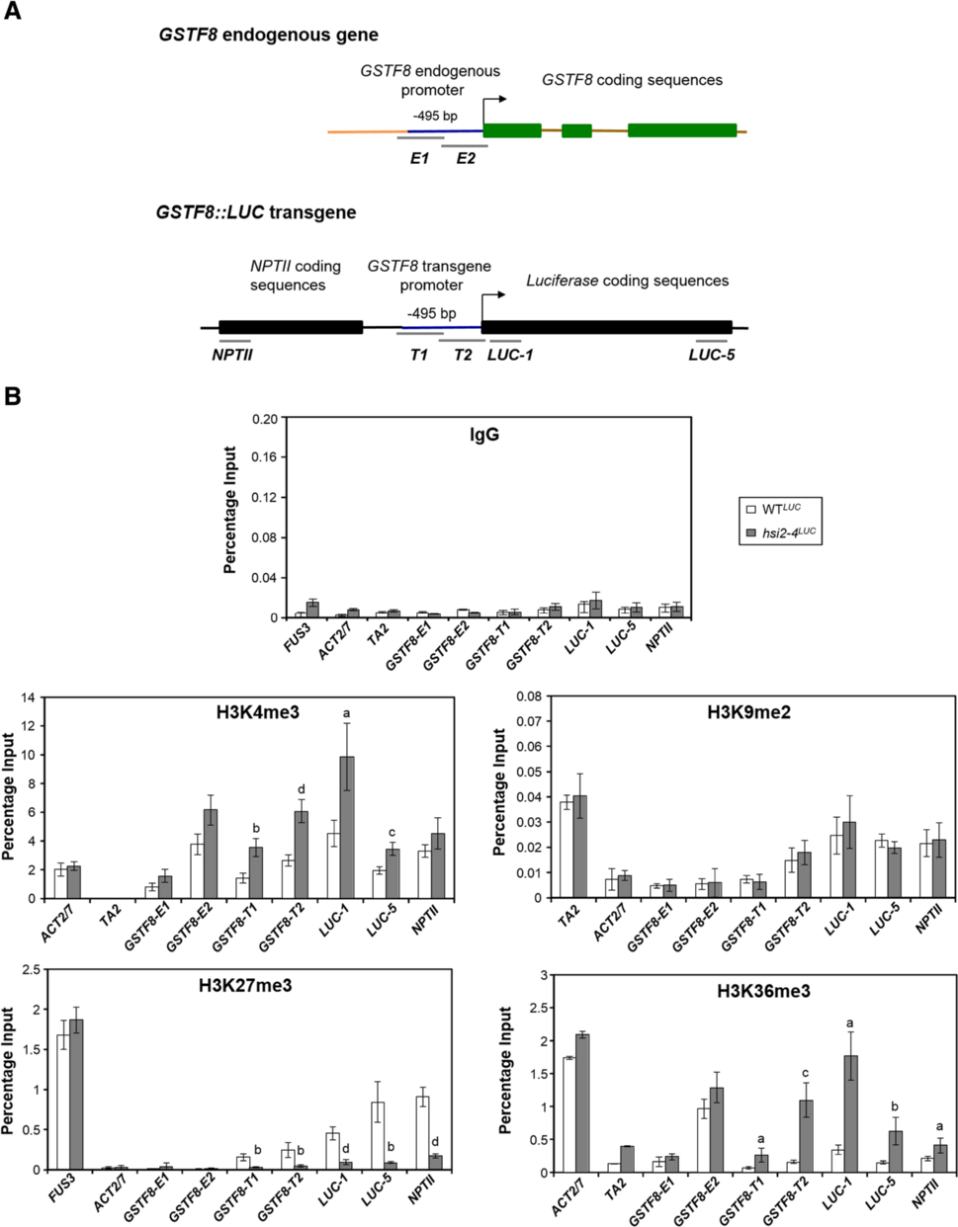
**Chromatin immunoprecipitation (ChIP) and quantitative PCR (qPCR) analyses of H3K4me3, H3K9me2, H3K27me3 and H3K36me3 levels on endogenous** ***GSTF8*** **promoter and transgene chromatin in WT** ^***LUC***^ **and** ***hsi2-4*** ^***LUC***^ **mutant. A**. Genomic structures of endogenous *GSTF8* gene and *GSTF8::LUC* transgene showing the locations of amplified regions by ChIP-qPCR. **B**. qPCR analyses of chromatin samples from 5-day old seedlings of WT^*LUC*^ and *hsi2-4* ^*LUC*^ that were immunoprecipitated using either specific antibodies recognizing indicated histone methylation marks or IgG (non-specific binding control). Data is expressed as percentage of immunoprecipitated DNA relative to input DNA. *ACT2/7* (H3K4me3 and H3K36me3), *FUS3* (H3K27me3) and *TA2* (H3K9me2) serve as positive controls whereas *TA2* (H3K4me3 and H3K36me3) and *ACT2/7* (H3K9me2 and H3K27me3) were used as negative controls. Data represent means (±SD) from two biological replicates with three qPCR replicates each. Significant differences in enrichment between WT^*LUC*^ and *hsi2-4* ^*LUC*^ for each genomic region tested were determined using two-tailed Student’s *t-*test assuming unequal variances and *P* values are indicated by letters (a = *p* < 0.05, b = *p* < 0.005, c = *p* < 0.0005, d = *p* < 0.0001).

To examine the histone methylation properties along the transgene cassette and the role of HSI2 PHD-like domain in regulating those marks and transgene expression, ChIP-qPCR analyses were performed using 5 day old seedlings of various genotypes. Antibodies specific to H3K4me3, H3K9me2, H3K27me3 and H3K36me3 marks were used, along with PCR primers that specifically amplify sequences from the endogenous (native) and transgene *GSTF8* promoters and *LUC* and *NPTII* coding regions. For the specific amplifications of *E1* and *E2* PCR fragments only from the endogenous *GSTF8* promoter sequence during ChIP-qPCR, at least one PCR primer that binds outside of the −495 bp endogenous *GSTF8* region that is not part of the *GSTF8::LUC* transgene cassette was used. Also, to make sure the PCR products of *T1* and *T2* fragments are only amplified from the *GSTF8* transgene promoter sequence, at least one primer that binds outside of the −495 bp region in the *GSTF8::LUC* transgene cassette was used ([14], Figure 5A). PCR amplification specificities of *E1*, *E2*, *T1* and *T2* fragments were confirmed using Col-0 wild-type and WT^*LUC*^. Among the histone methylation marks, H3K4me3 and H3K36me3 are associated with actively transcribed genes, while H3K9me2 is a repressive mark commonly enriched on transposable elements and repetitive sequences [24]. H3K27me3 is a repressive mark associated with transcribed genes that are under tissue-specific or developmental regulation [40–42]. Preimmune immunoglobulin G (IgG) was used as a negative control for non-specific binding and all genomic DNA fragments tested show very low background levels of enrichment when chromatin samples were immunoprecipitated with IgG (Figure 5B). *FUS3* was used as a positive control for H3K27me3, while actin2/7 (*ACT2/7*) was used as a negative control for H3K27me3 and as a positive control for H3K4me3 and H3K36me3. *TA2* was used as a positive control for H3K9me2 and as a negative control for H3K4me3 and H3K36me3. In agreement with our previous report [14], chromatin from the transgene *GSTF8* promoter region, and both 5′ and 3′ ends of the *LUC* coding sequences was highly enriched in H3K27me3 marks in WT^*LUC*^ seedlings (Figure 5B) while endogenous *GSTF8* promoter sequences showed consistently low levels of H3K27me3 (Figure 5B). Chromatin from the 5′ region of the *NPTII* coding sequence was also highly H3K27me3 enriched in WT^*LUC*^ seedlings (Figure 5B). A substantial decrease in H3K27me3 levels was detected on chromatin from the transgene *GSTF8* promoter sequences and *LUC* and *NPTII* coding sequences in *hsi2-4*^*LUC*^ seedlings that carry a point mutation in HSI2 PHD-like domain (Figure 5B). Though the transgene sequences tested showed considerable H3K9 dimethylation, unlike H3K27me3, no significant differences in H3K9me2 enrichment were seen between chromatin from WT^*LUC*^ and *hsi2-4*^*LUC*^ seedlings at any of the sites tested (Figure 5B). Therefore, among the histone methylation marks associated with transcriptional suppression, only H3K27me3 was dependent on the HSI2 PHD-like domain.

Histone methylation marks H3K4me3 and H3K36me3, which are associated with chromatin from actively transcribed genes, were enriched at all of the transgene sequences assayed in *hsi2-4*^*LUC*^ seedlings, relative to WT^*LUC*^ (Figure 5B). These marks were particularly abundant at the proximal transgene *GSTF8* promoter and 5′ *LUC* coding sequences but significant enrichment was also seen at the endogenous *GSTF8* promoter and *NPTII* coding sequence.

**Figure 6:**
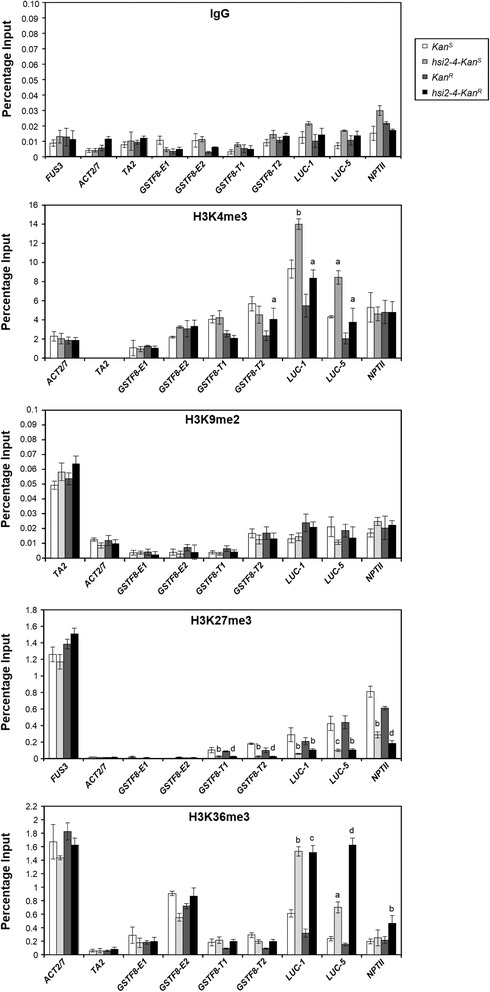
**ChIP-qPCR analyses of H3K4me3, H3K9me2, H3K27me3 and H3K36me3 enrichments on endogenous** ***GSTF8*** **promoter and** ***GSTF8::LUC*** **transgene regions in WT** ^***LUC***^ **and** ***hsi2-4*** **carrying either** ***Kan*** ^***S***^ **or** ***Kan*** ^***R***^ **locus.** Chromatin samples were prepared using five day old seedlings from various genotypes. ChIP-qPCR data represents mean values (±SD) of three PCR reactions obtained from each of two independent immunoprecipitations. Significant differences between wild-type lines and corresponding mutant lines (*Kan* ^*S*^ versus *hsi2-4-Kan* ^*S*^ *, Kan* ^*R*^ versus *hsi2-4-Kan* ^*R*^) were determined using two-tailed Student’s *t-*test assuming unequal variances and *P* values are indicated by letters (a = *p* < 0.05, b = *p* < 0.005, c = *p* < 0.0005, d = *p* < 0.0001).

To examine whether the *hsi2-4*-dependent changes in histone methylation marks are associated with both the *Kan*^*R*^ and *Kan*^*S*^ transgene loci, ChIP-qPCR analyses were performed on various regions of the endogenous *GSTF8* gene and the *GSTF8::LUC* transgene in *Kan*^*R*^, *Kan*^*S*^*, hsi2-4-Kan*^*R*^ and *hsi2-4-Kan*^*S*^ seedlings (Figure 6). As in chromatin from WT^*LUC*^ seedlings, higher levels of H3K27me3 marks at transgene *GSTF8* promoter sequences and at *LUC* and *NPTII* coding sequences were detected in wild-type seedlings carrying either the *Kan*^*R*^ or *Kan*^*S*^ transgene locus than in corresponding *hsi2-4-Kan*^*R*^ or *hsi2-4-Kan*^*S*^ mutant seedlings. Thus, the significant decrease in H3K27me3 levels at the *GSTF8::LUC* transgene associated with homozygosity for the *hsi2-4* allele was seen at both insertion sites. While chromatin from transgene sequences generally had higher levels of H3K9me2 marks than the endogenous *GSTF8* gene, no significant change was seen between these genotypes.

Enrichment of H3K4me3 and H3K36me3 was seen in chromatin at both *Kan*^*R*^ and *Kan*^*S*^ loci in *hsi2-4* seedlings (Figure 6). This enrichment was most pronounced at *LUC* coding sequences rather than in promoter regions and significant enrichment was also seen in chromatin of the *NPTII* gene at the *Kan*^*R*^ locus. Therefore, disruption of HSI2 PHD-like domain resulted in increased activation marks on 5′ and 3′ end of *LUC* coding sequences in both *Kan*^*R*^ and *Kan*^*S*^ backgrounds. However, increased H3K36me3 marks on the *NPTII* coding sequences were observed only in *hsi2-4-Kan*^*R*^ seedlings (Figure 6).

### H3K27me3 levels are significantly decreased on a subset of seed-maturation genes in *hsi2*^*LUC*^ mutant seedlings

**Figure 7:**
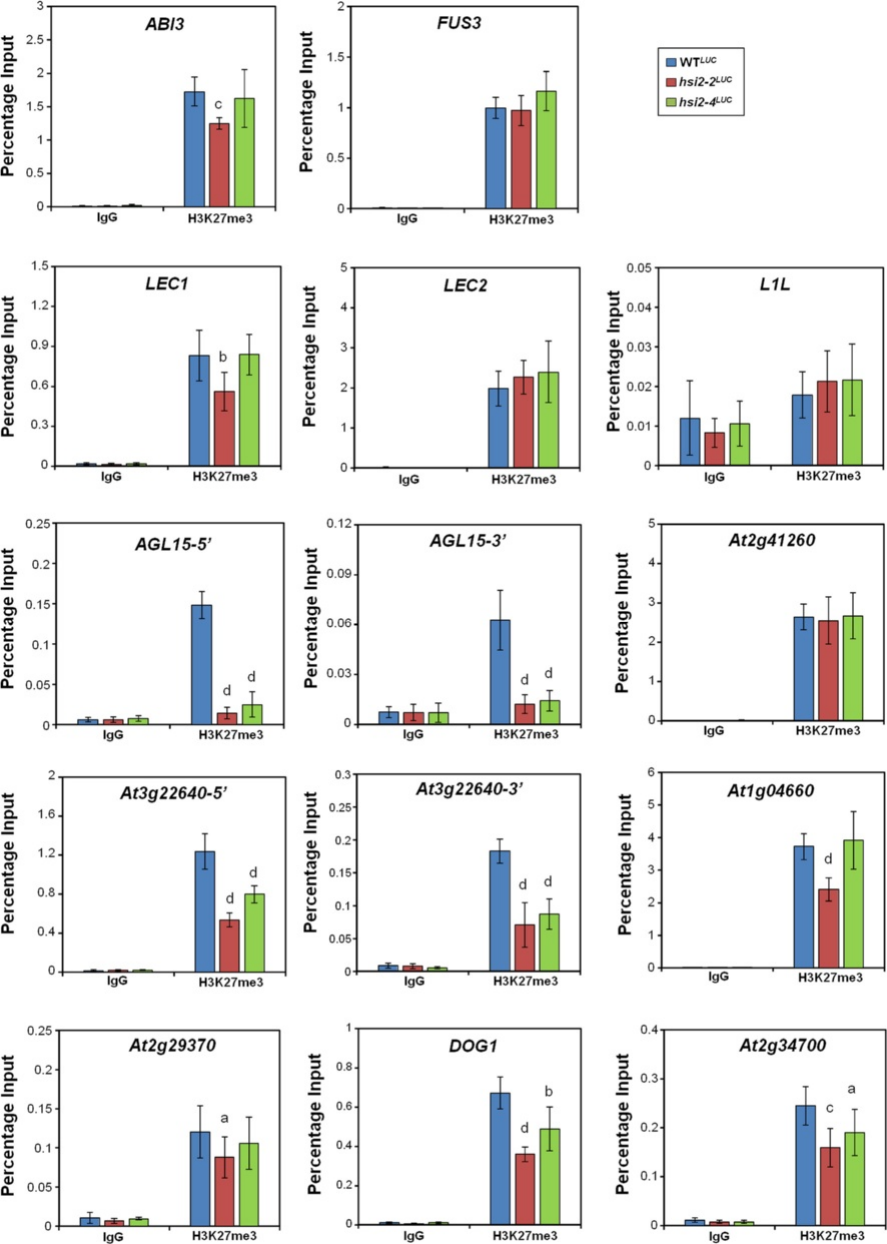
**ChIP-qPCR analyses of H3K27me3 levels on the master transcriptional regulators of seed-maturation program and seed maturation related target genes of HSI2 PHD-like domain in WT** ^***LUC***^ **and** ***hsi2*** ^***LUC***^ **mutant seedlings.** Chromatin samples were prepared using five day old WT^*LUC*^, *hsi2-2* ^*LUC*^ and *hsi2-4* ^*LUC*^ seedlings. Immunoprecipitations were performed using either IgG (non-specific binding control) or anti-H3K27me3 antibody. Data represent means (±SD) obtained from three qPCR reactions each from three independent immunoprecipitations from three different biological replicates. Significant differences in H3K27me3 enrichments between WT^*LUC*^ and *hsi2* ^*LUC*^ mutants (WT^*LUC*^ versus *hsi2-2* ^*LUC*^, WT^*LUC*^ versus *hsi2-4* ^*LUC*^) are indicated by letters (a = *p* < 0.05, b = *p* < 0.005, c = *p* < 0.0005, d = *p* < 0.0001). *ABSCISIC ACID-INSENSITIVE3 (ABI3,* At3g24650*), FUSCA3 (FUS3,* At3g26790*), LEAFY COTYLEDON1 (LEC1,* At1g21970*), LEAFY COTYLEDON2 (LEC2,* At1g28300*), LEC1-LIKE (L1L,* At5g4767*0), AGAMOUS-Like 15 (AGL15,* At5g13790*),* At2g41260 (Late-embryogenesis-abundant protein), At3g22640 (cupin family seed storage protein), At1g04660 (glycine-rich protein), At2g29370 (NAD(P)-binding Rossmann-fold superfamily protein), At5g45830 (*DOG1, DELAY OF GERMINATION1),* At2g34700 (putative proline-rich glycoprotein).

Some members of the LAFL clade of regulatory genes that control the expression of seed maturation genes [3] are misregulated in *hsi2* mutant seedlings [14]. *LEC1* and *ABI3* are ectopically expressed in *hsi2-2* but not in *hsi2-4* seedlings, while *FUS3* is upregulated in both *hsi2-2* and *hsi2-4* lines [14]. These results suggested to us that the HSI2-dependent negative regulation of *LEC1* and *ABI3* in seedlings does not require the PHD-like domain, while suppression of *FUS3* could be dependent on the PHD-like domain of HSI2. To determine if correlations exist between these expression patterns and histone modifications, ChIP-qPCR analysis of these genes was carried out on chromatin samples from WT^*LUC*^, *hsi2-2*^*LUC*^ and *hsi2-4*^*LUC*^ seedlings using antiH3K27me3 (Figure 7). Consistent with our hypothesis, significant reductions in H3K27me3 chromatin marks were detected in association with *ABI3* and *LEC1* genomic sequences only in chromatin from *hsi2-2*^*LUC*^ but not *hsi2-4*^*LUC*^ mutant seedlings and genes such as *LEC2* and *L1L*, which are not misregulated in either *hsi2* mutant allele alone, also showed no change in H3K27me3 marks in these mutant backgrounds. However, H3K27me3 marks associated with *FUS3* sequences were not altered in either mutant background. Thus, the effects of HSI2 on *FUS3* expression do not appear to depend on alterations in H3K27me3. On the other hand, the enrichment of H3K27me3 marks detected on both 5′ and 3′ coding sequences of *AGL15* (At5g13790) in WT^*LUC*^ seedlings was significantly decreased in both *hsi2-2*^*LUC*^ and *hsi2-4*^*LUC*^ backgrounds, which is consistent with the increased expression of *AGL15* in these mutants [14]. Therefore, the HSI2 PHD-like domain does appear to be required for both the repressed expression and H3K27 hypermethylation of the *AGL15* locus.

As previously reported, a number of seed maturation-related structural genes are derepressed in *hsi2-4* mutant seedlings [14]. To investigate whether the increased expression of these genes in *hsi2* mutant seedlings correlates with changes in associated H3K27me3 marks, ChIP-qPCR analyses were performed on chromatin from WT^*LUC*^, *hsi2-2*^*LUC*^ and *hsi2-4*^*LUC*^ mutant seedlings using primers specific to At2g41260 (Late-embryogenesis-abundant (LEA) protein), At3g22640 (cupin family seed storage protein), At1g04660 (glycine-rich protein), At2g29370 (NAD(P)-binding *Rossmann*-fold superfamily protein), At5g45830 (*Delay of Germination1, DOG1*) and At2g34700 (proline-rich glycoprotein). These seed maturation genes were previously reported to be targets of H3K27 hypermethylation [41] and our results confirmed that H3K27me3 marks were enriched, relative to IgG, on the 5′coding sequences of these genes (Figure 7). In comparison to WT^*LUC*^*,* significant decreases in H3K27me3 enrichments were detected in both *hsi2-2*^*LUC*^ and *hsi2-4*^*LUC*^ mutant seedlings on chromatin associated with 5′ genomic sequences of *DOG1* and At2g34700, and at both 5′ and 3′ sequences of At3g22640 (Figure 7). On the other hand, H3K27me3 levels on At1g04660 and At2g29370 were significantly decreased only in the *hsi2-2*^*LUC*^ background and not in chromatin from *hsi2-4*^*LUC*^ seedlings (Figure 7). These data indicate that, as with the regulatory genes described above, derepression of seed maturation-specific gene expression in *hsi2* mutant seedlings often corresponds with decreased accumulation of H3K27me3 marks that may or may not depend on the presence of an intact HSI2 PHD-like domain. However, as with *FUS3*, the LEA-like protein gene At2g41260, which is expressed at elevated levels in both *hsi2-2* and *hsi2-4* mutant plants [14], is strongly enriched for H3K27me3 but these marks are not significantly reduced in either *hsi2* mutant.

## Discussion

Despite detailed genetic and functional characterization, the molecular mechanisms that underlie HSI2- and HSL1-mediated repression of seed maturation program in seedlings are still not fully understood [12–16]. HSI2 contains a PHD-like domain [3,12,14,26] and PHD domains can act as “readers” of the histone methylation status of target genes to regulate their expression [30]. Through characterization of a novel mutant allele, *hsi2-4*, which affects the expression of *GSTF8::LUC* transgenes and certain seed maturation genes [14], we provide evidence that the HSI2 PHD-like domain is involved in regulating the expression of some genes by altering histone modifications.

Quantitative PCR data indicates that both *Kan*^*R*^ and *Kan*^*S*^ transgene loci in the WT^*LUC*^ reporter gene line contain multiple copies of the *GSTF8::LUC* transgene. The *Kan*^*R*^ locus is more complex than *Kan*^*S*^, harboring five copies of the transgene, while the *Kan*^*S*^ locus includes two (Table 1). Transgene loci in plants that harbor multiple and complex transgene repeats at a single locus were frequently targeted by DNA methylation-associated H3K9me2 marks and also histone deacetylation mediated transcriptional gene silencing [35,43–45]. However, treatment of WT^*LUC*^ seedlings with either the DNA methylation inhibitor 5-azadC or the histone deacetylase inhibitor TSA failed to derepress the *LUC* expression in WT^*LUC*^ seedlings (Figure 3A and B). Also, ChIP-qPCR analyses showed no differences in DNA methylation-associated H3K9me2 histone methylation marks on transgene sequences between WT^*LUC*^ and *hsi2-4* mutants that harbor either individual *Kan*^*R*^ and *Kan*^*S*^ loci or both (Figures 5B and 6). Based on these data, it appears that DNA methylation and histone deacetylation mechanisms are not involved in HSI2 PHD-like domain-mediated repression of transgene expression in WT^*LUC*^ seedlings.

Although 5-azadC did not affect *LUC* expression in WT^*LUC*^ seedlings (Figure 3A), *hsi2* mutant seedlings treated with various concentrations of 5-azadC maintained root and hypocotyl growth better than WT^*LUC*^ seedlings under the same conditions (Figure 4A and B). These results could indicate that HSI2 is somehow involved in the inhibition of seedling growth and development caused by 5-azadC and, since this effect was observed with both the PHD-like domain mutant allele *hsi2-4* and the *hsi2-2* T-DNA knock-out allele, it appears that the PHD-like domain may be required for this 5-azadC-dependent inhibition of growth. *LEC1*, an HSI2 and HSL1 target gene and member of the “LAFL network”, was shown to be regulated by DNA methylation [46,47] and the embryonic phenotypes of gain-of-function *lec1* mutants were enhanced by treatment with a DNA methylation inhibitor 5-azacytidine [46]. Hence, the partial rescue of seedling growth in *hsi2* mutants in the presence of 5-azadC could be an indirect effect of changes in the DNA methylation status of HSI2-targeted regulatory genes, including *LEC1*.

Data presented here clearly indicate that the HSI2 PHD-like domain is involved in suppressing the expression of *GSTF8::LUC* transgenes in both *Kan*^*R*^ and *Kan*^*S*^ transgene loci (Figure 2). Furthermore, the levels of *LUC* expression seen in *hsi2-4* plants that carry these reporter complexes correlate with transgene copy number, with relatively low levels of *LUC* expression seen in *hsi2-4*, *Kan*^*S*^ plants that contain two *GSTF8::LUC* copies and correspondingly higher levels expression in *hsi2-4, Kan*^*R*^ or *hsi2-4*^*LUC*^ lines with five and seven total reporter gene copies, respectively. The direct correlation between derepressed *LUC* expression and the number of reporter gene copies means that the luminescence of mutant plants with compromised HSI2-dependent repression will be amplified in a high copy number reporter gene line, making these mutants far more apparent in a luminescence-based mutant screen. On the other hand, *LUC* expression in wild-type plants that carry the *Kan*^*R*^ locus alone showed higher levels of *LUC* transcripts than did WT^*LUC*^ seedlings (Figure 2). These results appear to indicate that the presence of both *Kan*^*R*^ and *Kan*^*S*^ loci in the same genome may lead to stronger transcriptional suppression of the *GSTF8::LUC* reporter genes than when the *Kan*^*R*^ locus is present alone. No *NPTII* transcripts were detected in either *Kan*^*S*^ or *hsi2-4*-*Kan*^*S*^ seedlings (Figure 2), which is consistent with the kanamycin sensitivity of these plants. However, *hsi2-4-Kan*^*R*^ seedlings showed 3-fold higher expression of *NPTII* transcripts relative to wild-type *Kan*^*R*^ seedlings but this was not seen in corresponding *hsi2-4*^*LUC*^ and WT^*LUC*^ seedlings (Figure 2). Thus, as with the *LUC* reporter gene, the co-existence of *Kan*^*R*^ and *Kan*^*S*^ loci is associated with stronger transcriptional repression of *NPTII* gene expression. To better understand the function of the HSI2 PHD-like domain, interactions between activation-associated and repressive histone methylation marks at the *Kan*^*R*^ and *Kan*^*S*^ transgene loci were evaluated by ChIP-qPCR assays. Transgene *GSTF8* promoter sequences, along with *LUC* and *NPTII* coding sequences were highly enriched in H3K27me3 marks in WT^*LUC*^ seedlings (Figures 5B and 6) and significantly lower amounts of H3K27me3 were observed on these transgene sequences in the *hsi2-4* mutant background (Figure 5B). Similar histone modification patterns were observed in seedlings harboring individual *Kan*^*R*^ or *Kan*^*S*^ loci (Figure 6). Thus, the PHD-like domain of HSI2, which is required to repress the expression of these transgene complexes, is also necessary for the appearance of H3K27me3 marks on these loci. In contrast, H3K4me3 and H3K36me3 histone methylation marks, which are associated with active gene expression and have been shown to inhibit H3K27me3 marks on transcribed genes in both animals and plants [48–50], were enriched on these transgene sequences in *hsi2-4* seedlings, relative to those with the wild-type *HSI2* allele. Thus, the decrease in H3K27me3 marks on transgene sequences in both *Kan*^*R*^ and *Kan*^*S*^ loci in the *hsi2-4* mutant background is associated with both increased expression (Figure 2) and increased accumulation of H3K4me3 and H3K36me3 marks (Figures 5B and 6). Developmental repression of transcribed genes is often associated with H3K27me3 marks [41,42] but emerging evidence also suggests that H3K27me3 may act as an alternative to DNA methylation-associated H3K9me2 in transposable elements and repetitive sequence silencing [40,51–54]. Turck et al. [40] showed that the chromodomain-containing H3K27me3 “reader” protein LHP1 (LIKE HETEROCHROMATIN PROTEIN 1) is enriched on tandemly duplicated genes, such as the nine closely linked chitinase/glucosylase-18 genes (At4g19720-At4g19820) on chromosome 4 of Arabidopsis, but not on segmentally duplicated genes. Expressed genes that flank tandemly duplicated gene loci are also not associated with LHP1. Both *Kan*^*R*^ and *Kan*^*S*^ loci contain multiple *GSTF8::LUC* transgenes at individual loci (Table 1) and expression of genes that flank the *Kan*^*R*^ transgene locus does not differ between WT^*LUC*^ and *hsi2-4*^*LUC*^ seedlings [14]. However, similarities in *HSI2*-dependent H3K27me3 accumulation at the *Kan*^*R*^ and *Kan*^*S*^ loci and the corresponding relative changes in *LUC* expression in *hsi2-4* plants lead us to speculate that transcriptional repression is mediated by the *GSTF8::LUC* transgene itself and is not dependent on tandem T-DNA insertions. The presence of multiple transgene copies results in high levels of expression that accentuates the apparent repressive effect of HSI2 when its activity is compromised by mutation. However, since *LUC* expression in WT^*LUC*^ seedlings is lower than in *Kan*^*R*^ seedlings, the presence of these two unlinked loci appears to have synergistic effects on reporter gene silencing.

In contrast to the *GSTF8::LUC* reporter genes, expression of native *GSTF8* transcripts derived from either the “long” or “short” transcriptional start sites show no significant increase in the *hsi2-4*^*LUC*^ mutant background (Figure 2). The most parsimonious explanation for the discrepancy between native *GSTF8* expression and the expression of the *GSTF8::LUC* reporter gene is that the isolated *GSTF8* promoter sequence used in the *GSTF8::LUC* gene construct, which corresponds with the short promoter as defined by Thatcher et al. [34], could contain *cis*-acting suppressor elements that are masked in the context of the native gene. Support for this explanation can be seen in Figure 4B of Veerappan et al. [14]. In this experiment, LUC expression (measured as luminescence) was assayed in WT Col-0 and mutant *hsi2-4* Arabidopsis plants newly transformed with either short*-GSTF8::LUC* or long*-GSTF8::LUC* gene constructs (Col-S, Col-L and *hsi2-4-*S and *hsi2-4-*L, respectively). No significant differences in luminescence were apparent between Col-L and *hsi2-4*-L plants but LUC expression driven by the short *GSTF8* promoter in *hsi2-4-S* plants was substantially elevated relative to that detected in Col-S plants.

Polycomb group (PcG) proteins are evolutionarily conserved multi-protein complexes required for developmental repression of gene expression by chromatin based mechanisms. PcG proteins in plants comprise of two major complexes: Polycomb Repressive Complex 1 (PRC1) and PRC2 [4–6,55]. Arabidopsis PRC1 proteins BMI1 and RING1 were shown to have histone H2A mono ubiquitination (H2Aub) activity *in vitro* [16,56,57], whereas PRC2 complex proteins catalyze the deposition of H3K27me3 marks to promote developmental repression in animals and plants [58–60].

The *GSTF8* promoter sequence used in the *GSTF8::LUC* reporter contains an *octopine synthase* (*OCS)* sequence element at −460 (Additional file 1) that is known to be required for transcriptional activation in response to a variety of biotic and abiotic stress signals [61]. This *OCS* element is flanked by *OCS element binding factor 5 (OBF5)*and *OCS element binding proteins 1 (OBP1)* elements that were shown to bind proteins of the *DNA binding with One Finger (DOF)* family of transcription factors and are reported to act either as positive or negative regulatory factors in various plant genes [62]. A putative *myeloblastosis2* (*MYB2*) binding element is also located at −311 but its potential function is unknown. Sequence elements with a potential role in PRC2-based silencing can also be identified in the *GSTF8* promoter. By analyzing the co-distribution of the ubiquitous Arabidopsis PRC2 protein FERTILIZATION INDEPENDENT ENDOSPERM (FIE) and H3K27me3 marks, Deng et al. [63] identified four sequence motifs that could act as PRC2 binding sites. Two of these motifs are found in the *GSTF8* promoter, a putative GAGA box located between −123 and −131 relative to the transcription start site and a TTC repeat element located in the 5′ untranslated region between +36 and +56. GAGA elements were found to be specifically associated with FIE and H3K27me3 enriched sites and these sequences are also found in polycomb response elements (PREs) of Drosophila [63]. However, while GAA (reverse complement of TTC) sequence motifs were found to be associated with genomic regions that bind both FIE and H3K27me3, they were also enriched in random promoter sequences and were, therefore, not considered to be specific PRC2 binding sites [63].

The role of the GAGA element in the transcriptional regulation of the PRC2-repressed *LEC2* gene was confirmed by Berger et al. [64]. However, mutational analysis showed that, in this context, the GAGA element acted as a required *cis*-activating element, which was associated with a distinct *cis*-repressing element, termed repressive LEC2 element (RLE) that apparently consists of two component sequences. Comparison of the *LEC2* RLE sequence with the *GSTF8* promoter identified a duplicated element identical to the 5′ component of the *LEC2* RLE. As in the *LEC2* gene, the putative *GSTF8* RLE-like sequence is located immediately downstream of the GAGA element (Additional file 1). Whether this putative GAGA-RLE motif plays a role in the transcriptional regulation of the *GSTF8* promoter in a transgene context is not known.

ChIP-qPCR analyses were carried out to investigate whether *LAFL* network genes and other seed maturation genes that are up-regulated in *hsi2* mutant seedlings are also associated with HSI2-dependent changes in H3K27me3 marks. With the exception of *L1L*, all the tested seed maturation genes are enriched with H3K27me3 marks in WT^*LUC*^ seedlings (Figures 7) in agreement with previous reports [41,58]. Significant reductions in H3K27me3 levels, relative to WT^*LUC*^ seedlings, were observed at some gene loci in both *hsi2-2*^*LUC*^ and *hsi2-4*^*LUC*^ seedlings, while other genes showed reductions only in *hsi2-2*^*LUC*^ seedlings and a few showed no changes in either *hsi2* mutant background. In general, these HSI2-dependent differences in H3K27me3 marks correlate well with HSI2-dependent changes in gene expression. For example, among the regulatory genes tested, *ABI3* and *LEC1*, which are expressed at elevated levels in *hsi2-2* seedlings but not in *hsi2-4* seedlings [14], showed correspondingly decreased levels of H3K32me3 enrichment in chromatin from *hsi2-2*^*LUC*^ plants but not from *hsi2-4*^*LUC*^ plants (Figure 7). On the other hand, H3K27me3 marks on *AGL15* were strongly decreased in both *hsi2-2*^*LUC*^ and *hsi2-4*^*LUC*^ mutant plants (Figure 7) and expression of this gene is also upregulated in both *hsi2* mutant backgrounds. These results can be interpreted to indicate that HSI2-dependent transcriptional repression and H3K27 hypermethylation of *AGL15* is mediated by the PHD-like domain while that of *ABI3* and *LEC1* may be mediated by other HSI2 domains, such as the CW domain, which was shown to interact with HDA19 to promote histone deacetylation and H3K27me3 marks to repress seed maturation genes [31].

Similar patterns can be seen in the structural (non-regulatory) seed maturation gene sample. The putative glycine-rich protein gene (At1g04660) is more strongly expressed in *hsi2-2*^*LUC*^ plants than in *hsi2-4*^*LUC*^ plants [14] and H3K27me3 marks at this site are correspondingly reduced in *hsi2-2*^*LUC*^ but not *hsi2-4*^*LUC*^ mutants. On the other hand, genes for a cupin-like protein (At3g22640), *DOG1* (At5g45830) and a proline-rich glycoprotein (At2g34700) are similarly up-regulated in *hsi2-2*^*LUC*^ and *hsi2-4*^*LUC*^ plants and decreased H3K27me3 marks are also apparent in both mutant lines.

Two of the genes in our sample group do not show correlations between HSI2-dependent transcriptional repression and H3K27me3 marks. These genes, *FUS3* and the LEA protein gene At2g41260, are expressed at elevated levels in both *hsi2-2*^*LUC*^ and *hsi2-4*^*LUC*^ backgrounds and accumulate substantial H3K27me3 in WT^*LUC*^ plants but these marks are not diminished in either *hsi2* mutant. Therefore, derepression of these genes in *hsi2* mutant plants does not appear to require the depletion of H3K27me3. It seems likely that, in addition to the PRC2-mediated accumulation of H3K27me3 marks, other mechanisms are involved in the repression of these genes.

AGL15 is a member of the MIKC subfamily of MADS domain transcription factors that is preferentially expressed in developing embryos. Ectopic overexpression of *AGL15* results in enhanced somatic embryogenesis [65,66]. AGL15 acts upstream of *LAFL* network genes and several *LAFL* genes are direct regulatory targets of AGL15 [67,68]. *DOG1* is a seed-specific gene and plays a critical role in promoting seed dormancy by integrating environmental signals [69,70]. Recently, AGL15 and DOG1 were shown to be targets of H3K27me3 marks, which could be mediated by PRC1 proteins [58,71]. Our data shows that HSI2 regulates the expression of *AGL15* and *DOG1* in seedlings by promoting H3K27me3 marks possibly via PRC1-PRC2 complex which requires HSI2 PHD-like domain.

HSI2 was shown to directly interact with PRC1 complex proteins AtBMI1A/B/C and is required for the deposition of H2Aubi and H3K27me3 marks on “LAFL network” seed-maturation genes including *LEC1*, *FUS3* and *ABI3* [16]. Disruption of PRC2 complex genes in Arabidopsis led to decreased H3K27me3 levels, activation of “LAFL network” transcription factor genes and ectopic expression of embryonic traits during seed germination and vegetative development [58,72,73]. Thus, it is possible that HSI2 interacts with PRC1 proteins like AtBMI1 to recruit PRC2 proteins such as the histone methyltransferase CURLY LEAF (CLF), to deposit H3K27me3 marks on the *GSTF8::LUC* transgene loci. AtRING1a was also shown to physically interact with the PRC2 core component CLF [74] and several reports have demonstrated the involvement of PHD-PRC2 and PHD-PRC1-PRC2 complexes in deposition of H3K27me3 marks to promote transcriptional repression of gene expression in plants [16,71,75]. Similarly, HSI2 could be part of a repression complex that involves the HSI2 PHD-like domain, PRC1 and PRC2 complex proteins to promote high levels of H3K27me3 marks on native seed maturation genes and *GSTF8::LUC* transgene loci to repress their expression during the seed to seedling developmental phase transition.

## Conclusions

HSI2 contains a putative PHD domain, which could act as a “reader” of histone methylation marks. In this work, we show that HSI2 PHD-like domain regulates both *LUC* and *NPTII* transgenes from two independent transgene loci. Transcriptional repression of both of these transgene loci by HSI2 PHD-like domain is associated with repressive histone methylation marks H3K27me3 but not siRNA and DNA methylation associated H3K9me2 marks. In addition to the transgenes, HSI2 is also required for the repression of a subset of seed maturation genes in seedlings by promoting H3K27me3 marks in a PHD-like domain dependent and independent manner.

## Methods

### Plant materials, growth conditions and chemical treatments

*Arabidopsis thaliana* Columbia-0 (Col-0; CS60000) wild-type was obtained from Arabidopsis Biological Resources Center. WT^*LUC*^ and *hsi2-4*^*LUC*^, which contain both *Kan*^*R*^ and *Kan*^*S*^ transgene loci in Col-0 background were described before [14]. The other genotypes that were used in this study including *Kan*^*R*^, *Kan*^*S*^, *hsi2-4*-*Kan*^*R*^ and *hsi2-4*-*Kan*^*S*^ were obtained by crossing the WT^*LUC*^ and *hsi2-4*^*LUC*^ into the Col-0 wild-type. The *hsi2-2*^*LUC*^ line harbors *GSTF8::LUC* transgenes in the *HSI2* T-DNA knock-out allele *hsi2-2* (SALK_088606) background. For all the experiments described here, plants were grown under continuous illumination at 24°C on 0.3% Phytagel plates containing 0.5X Murashige and Skoog (MS) salt, 0.5 g/L MES (2-(N-morpholino) ethanesulfonic acid, 1X Gamborg vitamin mix and 1 % sucrose (pH adjusted to 5.7). 5-Aza-2′-deoxycytidine (5-azadC, A3656; Sigma) and Trichostatin A (T8552; Sigma) stocks were prepared using dimethyl sulfoxide and methanol respectively, and added directly to the MS media plates. Hypocotyl and root length measurements were made using Image J software (http://rsbweb.nih.gov/ij/). Digital photos of seedlings grown on 5-azadC plates were taken along with a ruler of known length. Hypocotyl length was measured from the tip of the apical meristem to the junction between hypocotyl and root, while root length was measured from the hypocotyl/root junction to the tip of the primary root.

### Luminescence imaging, genetic crosses and genotyping

Luminescence imaging was performed using Andor iKON-M DU934N-BV CCD camera (Andor Technology). After spraying with 1 mM D-luciferin potassium salt (Gold Biotechnology) containing 0.01% Triton X-100 solution, seedlings were kept in the dark for 5 minutes and imaging was performed with a 5 minute exposure. Andor SOLIS (I) imaging software (Andor Technology) was used for the acquisition of luminescence images and processing. To separate *Kan*^*R*^ and *Kan*^*S*^ loci, WT^*LUC*^ and *hsi2-4*^*LUC*^ mutant were crossed into Col-0 wild-type plants. Successful crosses were identified based on luciferase imaging in the F_1_ generation and plants were allowed to self-pollinate. Progeny lines homozygous for the *Kan*^*S*^ locus were identified based on kanamycin sensitivity whereas plants homozygous for the *Kan*^*R*^ locus were identified by PCR genotyping using T-DNA and genomic primers. To genotype *hsi2-4* mutation, a previously described CAPS marker [14] was used.

### Preparation of total RNA, cDNA synthesis and real-time reverse transcription quantitative PCR

Total RNA extraction and real-time reverse transcription quantitative-PCR (RT-qPCR) analysis was performed as described in Veerappan et al. [14]. Primers used in RT-qPCR are listed in Additional file 2.

### Estimation of *LUC* transgene copy numbers by real-time quantitative PCR

To determine the copy numbers of *LUC* transgenes in WT^*LUC*^, *Kan*^*R*^ and *Kan*^*S*^ lines, real-time quantitative PCR (qPCR) was performed as described before [32,33]. All qPCR reactions were performed using AB StepOnePlus Real-Time PCR System (Applied Biosystems) in 10 μl volume containing different amounts of DNA, 0.2 μM of each primers and 5 μl iTaqTM SYBR green supermix (Bio-Rad). Several sets of primers were tested for optimal performance. Temperature cycling conditions were 95°C for 10 minutes, 40 cycles for 15 seconds at 95°C and 1 minute at 60°C. Each DNA sample was tested in triplicates with three different DNA concentrations. Calibration curves were also performed in triplicates with five different DNA concentrations. Ct values were calculated using StepOne Software v2.1 (Applied Biosysytems). Concentrations of DNA samples were measured using Nanodrop 2000 (Thermoscientific) and the exact copy numbers of the template genome in the reactions were calculated using the following website: http://cels.uri.edu/gsc/cndna.html), applying the formula: number of copies = (amount * 6.022×10^23^)/(length * 1×10^9^ * 650). The calibration curves for *LUC* were created using the plasmid DNA pBI121-*GSTF8::LUC* as a template. At5g47480, a single copy gene from Arabidopsis, was used as an internal control for normalization of the data. PCR primers used in the estimation of transgene copy numbers can be found in Additional file 2.

### Chromatin immunoprecipitation and quantitative PCR analyses

Chromatin immunoprecipitation (ChIP) and quantitative PCR (qPCR) analyses were performed as described by Veerappan et al. [14]. Percentage of immunoprecipitated DNA relative to the total chromatin input was calculated for various samples using qPCR. Antibodies used for ChIP: normal rabbit IgG (Millipore, 12–370), anti-H3K4me3 (Millipore, 07–473), anti-H3K9me2 (Abcam, ab1220), anti-H3K27me3 (Millipore, 07–449) and anti-H3K36me3 (Abcam, ab9050). Primers used for ChIP PCR analyses are listed in Additional file 2.

## Additional files

Additional file 1:
**Analysis of** ***GSTF8*** **promoter sequence for the identification of putative** ***cis*** **-elements.**
Additional file 2:
**List of PCR primers used in this study.**

## Abbreviations

HSI2: High-level expression of sugar inducible gene2
HSL1: HSI2-Like1
VAL1: Viviparous1/ABI3-Like1
PHD: Plant homeodomain
LUC: Luciferase
5-azadC: 5-aza-2′-deoxycytidine
GSTF8::LUC: Glutathione *S*-transferase F8::luciferase
NPTII: Neomycin phosphotransferase II
LEC1: Leafy cotyledon1
LEC2: Leafy cotyledon2
L1L: LEC1-Like
ABI3: Abscisic acid insensitive3
FUS3: FUSCA3
LAFL: AFL (ABI3/ FUS3/ LEC2)/LEC (LEC1/L1L)
GUS: β-glucuronidase
CW: Cysteine and tryptophan residue-containing
EAR: Ethylene-responsive element binding factor-associated amphiphilic repression
siRNAs: Small interfering RNAs
ChIP: Chromatin immunoprecipitation
qPCR: Quantitative PCR
WT^LUC^: Wild-type luciferase
Kan^R^: Kanamycin resistant
Kan^S^: Kanamycin sensitive
AGL15: AGAMOUS-Like15
GSTF8-L: GSTF8-long
GSTF8-S: GSTF8-short
TSA: Trichostatin A
ACT2/7: Actin2/7
IgG: Immunoglobulin G
H3K27me3: Trimethylation of histone H3 at lysine 27
H3K4me3: Trimethylation of histone H3 at lysine 4
H3K36me3: Trimethylation of histone H3 at lysine 36
H3K9me2: Dimethylation of histone H3 at lysine 9
LEA: Late embryogenesis abundant
DOG1: Delay of germination1
LHP1: Like heterochromatin protein1
PcG: Polycomb group
PRC1: Polycomb repressive complex 2
PRC2: Polycomb repressive complex 1
H2Aub: H2A mono ubiquitination
OCS: Octopine synthase
OBF5: OCS element binding factor 5
OBP1: OCS element binding proteins 1
DOF: DNA binding with one finger
FIE: Fertilization independent endosperm
MYB2: Myeloblastosis2
PREs: Polycomb response elements
RLE: Repressive LEC2 element
CLF: Curly leaf
