# Supplementary material for: HSI2/VAL1 PHD-like domain promotes H3K27 trimethylation to repress the expression of seed maturation genes and complex transgenes in Arabidopsis seedlings
Source: BMC Plant Biol. 2014 Nov 1;14:293. doi: 10.1186/s12870-014-0293-4 (PMC4232687; doi:10.1186/s12870-014-0293-4)
Supplement: Additional file 1: — Analysis of GSTF8 promoter sequence for the identification of putative cis -elements. [file 12870_2014_293_MOESM1_ESM.pdf]

```
                    OBF5         OCS                OBP1
-481 TCTAGAATCTTATGTCATTGATGACGACCTCCTCTACACTTTTGGATTTG

-431 TATGTTTACAAAGGAAGGAAGGATTACCTTAGAACAGTTTATGTGATGTT

-381 TGTGTCTTTCTATTTTTATTCAATGAAATTTCTTTACATGTACATAGATT
                           MYB2
-331 AGCTATATTGACATGAATCTAACTGATTTTTTTGTTTGCAAAATAACAAA

-281 TTAGTGAGGGGAATTTTGTTTCAGTCAATAAATCCGTGATTTCTATATGT

-231 TAGATGGATAACAATGCAATAAATAAAGGGGACCCAATAAAAAAAAAGGA
      GAGA box         LEC2-like RLE
-131 AAGAGAGACGTGCCTGTGGGTAGTGGGTGGTGGTGAGAAGATTCTGGAAA
                                                  TATA
-81  AATGGGAGCAATTCAAGCTCGTCTTCCCTTGTTCCTTTCTCCCCCTTCTA
     box                           +1
-31  TAAAACACCATACCTTCCTTCATTCTTCTTCATCCAATTCCAATTTCAAA
                               GAA motif         Start codon
+20  ATCAGATCCAACAAATCTTCTTCTTCTTCTTCTTCTTCGATCATCATG
```

Additional file 1. Analysis of *GSTF8* promoter sequence for the identification of putative *cis*-elements.
