# Supplementary material for: HSI2/VAL1 PHD-like domain promotes H3K27 trimethylation to repress the expression of seed maturation genes and complex transgenes in Arabidopsis seedlings
Source: BMC Plant Biol. 2014 Nov 1;14:293. doi: 10.1186/s12870-014-0293-4 (PMC4232687; doi:10.1186/s12870-014-0293-4)
Supplement: Additional file 2: — List of PCR primers used in this study. [file 12870_2014_293_MOESM2_ESM.pdf]

Additional File 2. List of PCR Primers used in this study

qRT-PCR Primers (5'-3')

GSTF8-Long-F- GACGTGCCTGTGGGTAGTG

GSTF8-Long-R- GGTGTTTTATAGAAGGGGGAGA

GSTF8-Total-F-CTCTTTGACTCTCGCCCTAAGG

GSTF8-Total-R-AGCAGGCCTGGCAGAGATC

LUC-1-F-ATTTATCGGAGTTGCAGTTGCGCC

LUC-1-R-GCTGCGAAATGCCCATACTGTTGA

NPTII-F-ACCTTGCTCCTGCCGAGAAAGTAT

NPTII-R- ATGTTTCGCTTGGTGGTCGAATGG

qPCR Primers used in transgene copy number estimation (5'-3')

LUC-5F- GCCAGTCAAGTAACAACCGCGAAA

LUC-5R- CTTTCCGCCCTTCTTGGCCTTTA

At5g47480-F-CCGACACCATCTCCAATTGCAA

At5g47480-R-TACTGCTACTGTTCAGTGGGCTTG

ChiP-PCR Primers (5'-3')

ABI3-F-GAGATCTGGCTGAGGATTGT

ABI3-R-CTCTACCAACTTCATCAATTCCATC

FUS3-F-AGTTGGCACGTGGGAAATAG

FUS3-F-GTGGCAAGTGTTGATCATGG

LEC1-F-TGAACTTGGACCAGCACAG

LEC1-R-CTGGACCACGATACCATTGTT

LEC2-F-ACTTCTTACCCTTTCCCTCTTC

LEC2-R-GTGCGAGCGATTGTTGTTAG

L1L-F- TGAACAACACCACTCCTTCTC

L1L-R-TGAATTCTGGAGGACGCATAC

AGL15-5'-F-TCGAGATAAAGAGGATCGAGAATG

AGL15-5'-R-GCAACTTCAGCATCACAAAGA

AGL15-3'-F-GAAGGACGAATGAAGGAGAAAGA

AGL15-3'-R-CTTCAGGTGGAGAATTTGCTAAAC

At2g41260-5'-F-ACCTCAAGTCTCTCGTTCTCT

At2g41260-5'-R-GCTTCAGTACTTGGCTTCACT

At3g22640-5'-F-CACTTCTTCTCCTTATCTCCATAGC

At3g22640-5'-R-GGGACCTTCTTGTTCTCCTTG

At3g22640-3'-F-ACTAGGGTAACCTTTGGTGTTG

At3g22640-3'-R-CTCGTAGGTGCAAAGTAGGAATAG

At1g04660-5'-F-TGTGTCTTGCTCGCCATATC

At1g04660-5'-R-GACGCTCTTTGGTGGTAACT

At2g29370-5'-F-AGGCATGACTGCTCTTGTTAC

At2g29370-5'-R-CTGGCACATGTGTGAACTCT

DOG1-5'-F-GGATCTTCATCAAAGAACATCGAAC

DOG1-5'-R-TCAGGGATGCGTTGAGATTG

At2g34700-5'-F-AGCTCTCAAATATCTCTTACTCCTTTC

At2g34700-5'-R-GTCATTGGAGAAGCGGATGAA

TA2-F-AAACGATGCGTTGGGATAGGTC

TA2-R-TACTCTCCACTTCCCGTTTTTCTTTTTA

ACT2/7-F-CCCTCGTAGATTGGCACAGT

ACT2/7-R-GGCCGTTCTTTCTCTCTATGC

GSTF8-T1-F-GCTATGACCATGATTACGCCAAGC

GSTF8-T1-R-GAAATCACGGATTTATTGACTGAAACA

GSTF8-T2-F-TCCGTGATTTCTATATGTTAGATGGA

GSTF8-T2-R-TACCAACAGTACCGGAATGCCA

GSTF8-E1-F-GAGATTCGTTTCTAGGAGAGGTTGAC

GSTF8-E1-R-TCACGGATTTATTGACTGAAACA

GSTF8-E2-F-TGTTTCAGTCAATAAATCCGTGA

GSTF8-E2-R-GAACTCCGTGAACCTTGATACTGG

LUC-1-F-ATTTATCGGAGTTGCAGTTGCGCC

LUC-1-R-GCTGCGAAATGCCCATACTGTTGA

LUC-5-F-GCCAGTCAAGTAACAACCGCGAAA

LUC-5-R-TCTTTCCGCCCTTCTTGGCCTTTA

NPTII-F-ACCTTGCTCCTGCCGAGAAAGTAT

NPTII-R- ATGTTTCGCTTGGTGGTCGAATGG
